# Comparison of DN4 and DN4 Interview Tests in the Identification of Neuropathic Pain after Fracture Surgery

**DOI:** 10.1055/s-0044-1779686

**Published:** 2024-03-21

**Authors:** Gustavo Waldolato, Glauciana de Sousa Pereira, Isabela Storch Carvalho, Janaine Cunha Polese, Amanda Aparecida Oliveira Leopoldino

**Affiliations:** 1 Departamento de Ortopedia, Hospital Universitário Ciências Médicas de Minas Gerais, Belo Horizonte, Minas Gerais, Brasil; 2 Programa de Pós-Graduação em Ciências da Reabilitação, Universidade Federal de Minas Gerais, Belo Horizonte, Minas Gerais, Brasil

**Keywords:** complex regional pain syndromes, fracture fixation, interview, telephone, pain, surveys and questionnaires

## Abstract

**Objective:** This study aimed to compare results obtained with the DN4 (in-person interview) and DN4i (telephone interview) questionnaires in identifying neuropathic pain after fracture surgery.

**Methods:** This study was methodological, using questionnaires administered in person (DN4) or via telephone (DN4i). The participants were at least 18 years old, underwent fracture surgery at a university hospital between January 2017 and July 2020, signed the Informed Consent Form (ICF), and could go to the Orthopedics and Traumatology Hospital. Pearson's correlation coefficient determined the agreement between the total score obtained during in-person and telephone interviews. The kappa coefficient evaluated the agreement between individual questionnaire items.

**Results:** Of the 53 participants, 50 presented the same result for neuropathic pain screening in DN4 and DN4i, including 41 with a positive score for neuropathic pain and 12 with a negative score. The Pearson's correlation coefficient and kappa coefficient were r = 0.84.

**Conclusion:** DN4 and DN4i presented a strong agreement between individual items of the questionnaires and the total scores obtained.

## Introduction

Neuropathic pain is “a pain arising as a direct result of an injury or disease affecting the somatosensory system.” 1 Neuropathic pain-associated factors include the female gender and advanced age. 2 Recent studies also reported that fracture and surgery are a common etiology for neuropathic pain. 3 4

Although epidemiological data on neuropathic pain remains not detailed in the literature, it has evolved in recent years. 5 In Brazil, its estimated prevalence in patients with chronic pain and neuropathic characteristics is 14.5%. 6

Among the tools for neuropathic pain screening, the *Douleur Neuropathique* 4 (DN4) questionnaire is more practical than others as it has fewer items and a high capacity to discriminate neuropathic pain from nociceptive pain. 7 8 DN4, developed by the French neuropathic pain group, contains ten items answered as yes or no; a score equal to or higher than four indicates the presence of neuropathic pain. 9 In addition, the DN4 interview (DN4i) has the initial seven items alone, and a positive score is equal to or higher than 3. 9 Santos et al. 7 validated and translated the DN4 into Brazilian Portuguese.

Fast, highly sensitive tools for detecting neuropathic pain, whether used in person or not, allow a detailed determination of epidemiology and etiology, appropriate choice of therapeutic interventions, and prognostic definition. Therefore, this study aimed to compare the results obtained through the in-person application of the DN4 questionnaire and the DN4i administered via telephone to identify neuropathic pain after fracture surgery.

## Materials and Methods

This study is methodological and based on medical records of patients undergoing fracture surgery in our hospital from January 2017 to July 2020. The Research Ethics Committee approved this study under number (CAAE: 28504919.5.0000.5134).

This study used a convenience sample and recruited all patients meeting the eligibility criteria, i.e., aged 18 or over undergoing fracture surgery in our hospital and who signed the Informed Consent Form (ICF) and could attend the hospital's Orthopedics and Traumatology department. Patients who refused to participate in the study, did not answer the phone call, or died were excluded.

A previously trained group of orthopedists from our clinical staff applied the validated DN4 translated into Brazilian Portuguese to patients in person. After 4 to 6 months, two researchers unaware of the results obtained by orthopedists administered the DN4i questionnaire by telephone to their respective patients. All collected data was organized in an Excel spreadsheet for later analysis.

The DN4 questionnaire has two parts. The first part is an interview with seven items, while the second is a sensory examination with three items. DN4i contains only the interview, allowing self-application by the patient or telephone use. The seven-item part encompasses two domains: the first evaluates the characteristics of the pain (burning, painful cold sensation, and electric shock), and the second assesses symptoms associated with abnormal sensations in the same area (tingling, pins and needles, numbness, and itching). The second part of DN4, the sensory examination, addresses hypoesthesia to touch, hypoesthesia to needle prick, and pain caused or increased by brushing. Each item in both questionnaires is answered as “yes” (equivalent to 1) or “no” (equivalent to 0). The sum of the scores can range from 0 to 10 for DN4, and from 0 to 7 for DN4i. A score positive for neuropathic pain corresponds to a sum ≥ 4 for DN4 and ≥ 3 for DN4i. 9

An independent investigator performed the analysis. Descriptive statistics characterized the sample considering all variables collected. These data were presented as measures of central tendency (mean/median) and dispersion (standard deviation) for quantitative variables and frequency and percentage for categorical variables.

The Pearson's correlation coefficient investigated the agreement between the total questionnaire score obtained in person (DN4) and by telephone (DN4i). Considering the statistically significant data, the interpretation of the magnitude of the correlation coefficient occurred as follows: strong (r ≥ 0.6), moderate (r ≤ 0.59), and weak (r ≤ 0.29). 10

The weighted kappa statistic determined the agreement between individual questionnaire items. The interpretation of kappa occurred as follows: excellent (> 0.80), substantial (> 0.60), moderate (0.40-0.60), and fair to poor (< 0.40). 11 The SPSS 17.0 statistical software for Windows performed all analyses using a 5% significance level.

## Results

**Fig. 1:**
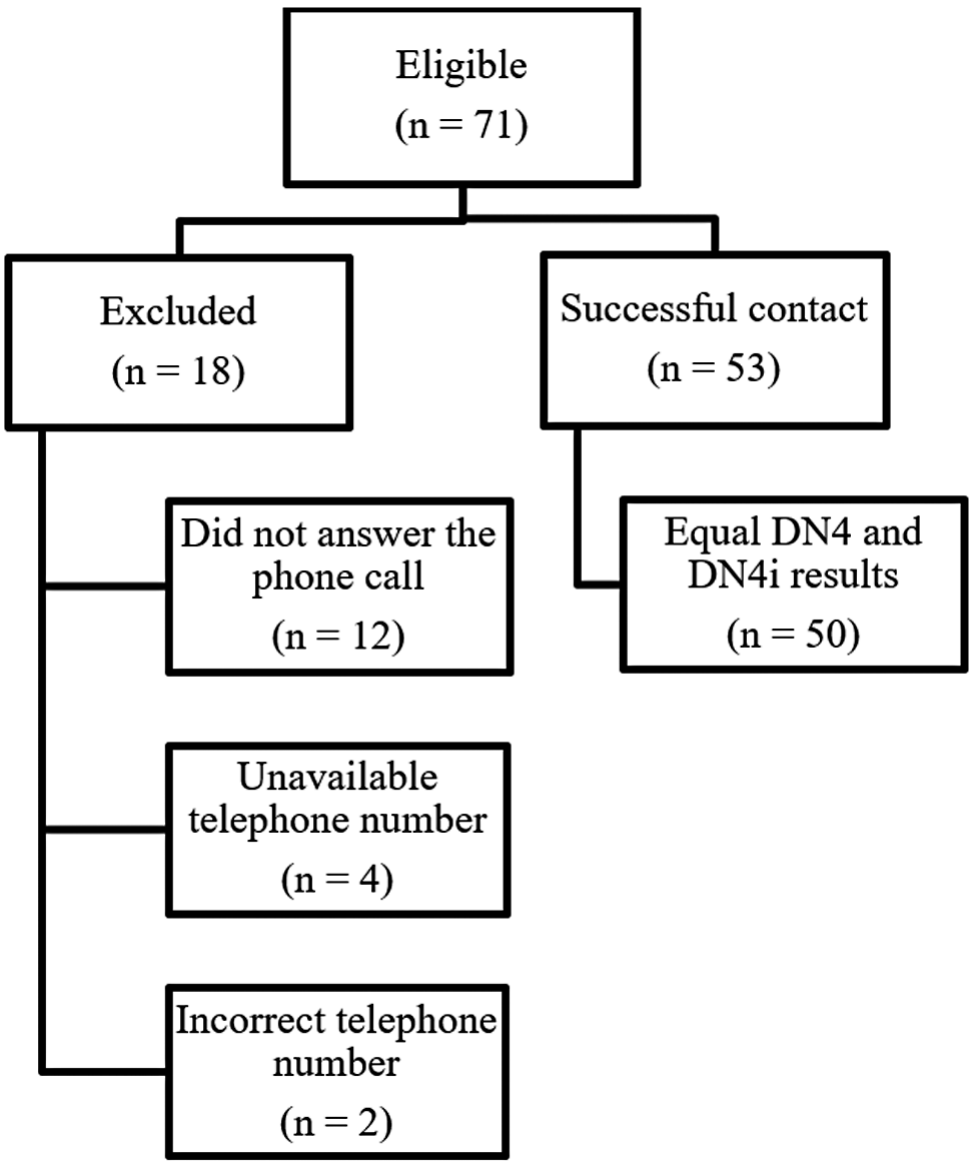
Sample recruitment according to eligibility criteria.

In total, 71 participants were eligible for the study. The final sample had 53 participants with analyzable data, of which 50 (94.3%) presented the same results in DN4 and DN4i for neuropathic pain screening. Fig. 1 shows the sample layout.

Table 1 shows the sociodemographic and clinical characteristics of the participants with analyzable data. Considering the Numerical Verbal Pain Scale, ranging from 0 to 10, 26.4% of the participants reported values between 2 and 8, of which half (13.2%) had a positive score on DN4i.

**Table 1:** Sociodemographic and clinical characteristics of the sample (n = 53)

With neuropathic pain (n = 12)	Without neuropathic pain (n = 41)
Female gender (n, %)	33 (62.3)
Mean age (±SD)	55.5 (± 17.7)
Mean BMI (±SD)	27.95 (±5.52)
Comorbidities (n, %)	33 (62.3)
Diabetes	11 (33.3)
Marital status (n, %)	
Single	21 (39.6)
Married	18 (34.0)
Widowed	10 (18.9)
Divorced	1 (1.9)
Not informed	3 (5.7)
Fracture site (n, %)	
Ankle	23 (43.4)
Radius	19 (35.8)
Proximal femur	11 (20.8)
Trauma mechanism (n, %)	
Fall from own height	23 (43.4)
Sprain	16 (30.2)
Fall from height	7 (13.2)
Traffic accident	6 (11.3)
Aggression	1 (1.9)
Numerical Verbal Pain Scale (n, %)	
Presence of pain	14 (26.4%)
Positive DN4i score	7 (13.2%)

The orthopedists administering DN4 in person obtained 13 positive (sum ≥ 4) and 40 negative scores, while the two researchers who made the telephone calls got 12 positive (sum ≥ 3) and 41 negative results. Table 2 compares the percentages of positive scores for DN4 and DN4i items.

**Table 2:** Comparison of positive response frequencies at DN4 and DN4i items (n = 53)

In-person interview (DN4)	n (%)
Burning	15 (28.3)
Painful cold sensation	5 (9.4)
Electric shock	9 (16.9)
Tingling	14 (26.4)
Pins and needles	12 (22.6)
Numbness	10 (18.9)
Itching	6 (11.3)
Hypoesthesia to touch	8 (15.1)
hypoesthesia to needle prick	4 (7.5)
Brushing	10 (18.9)
**Telephone interview (DN4i)**	
Burning	9 (16.9)
Painful cold sensation	8 (15.1)
Electric shock	8 (15.1)
Tingling	15 (28.3)
Pins and needles	8 (15.1)
Numbness	11 (20.8)
Itching	6 (11.3)

Pearson's correlation coefficient for agreement between the total questionnaire score was r = 0.84 (p < 0.001), deemed strong considering its magnitude. Regarding the agreement between the individual items of the questionnaires, the kappa coefficient was r = 0.84 (p < 0.001), which is excellent.

## Discussion

Seven of the 12 positive DN4i scores were from females, including four subjects with diabetes mellitus. This finding reinforces the factors associated with the development of neuropathic pain, such as female gender and diabetes mellitus as an associated comorbidity. 2 12 13

The most predominant fracture site was the ankle (n = 23), and 17.4% of the affected subjects had a positive score on DN4i. Rbia et al. 14 reinforce this finding, identifying the prevalence of neuropathic pain after ankle fracture surgery in 23% of 271 patients, impacting their quality of life.

Attal et al. 15 suggested doctors and healthcare professionals should use DN4i for fast neuropathic pain screening. A previous study from Bouhassira et al. 9 suggested the potential DN4i use in telephone surveys due to the significant discriminating properties of the seven items. This observation was based on the fact that DN4i's sensitivity (78%) and specificity (81.2%) are slightly lower than DN4 (82.9% and 89.9%, respectively). 9 16 However, VanDenKerkhof et al. 17 point out that test sensitivity may vary according to the clinical condition. These authors cited, for instance, a 92.5% sensitivity for central pain, while Aho et al. 18 reported a 66.2% sensitivity for peripheral nerve injury after a surgical procedure.

Despite the suggestion of telephone use of DN4i by Bouhassira et al., 9 few studies in the literature have analyzed it. One of them 16 validated the method during a complementary telephone survey, which had no reference, to determine the prevalence of chronic pain with neuropathic pain symptoms in a random populational sample from Alberta, Canada. Research participants received the call a week after the in-person application of DN4, which favors memory bias, as the patients could remember their answers and repeat them. Furthermore, the call was from the same doctor who applied DN4, compromising the internal and external validity of the study. In our study, different staff members performed the in-person application of the DN4 and the telephone interview using DN4i; however, these professionals had the same training, and the longer interval (4 to 6 months) could control the memory bias.

Keene et al. 19 corroborated that the use of the DN4i questionnaire via telephone months after DN4 application did not reduce the sensitivity for neuropathic pain screening in their multicenter study to identify the prevalence of neuropathic pain after lower limb fracture surgery through the application of DN4 in the third and sixth months. As a result, they observed that 10% of patients without neuropathic pain 3 months after surgery had pain at 6 months, which differs from the expectation that the intensity and character of pain improve with the time after the injury. 19

This study has some limitations, such as the difficulty in contacting patients who underwent surgery longer ago, which limited the number of participants. As it includes femur fractures, mortality bias in post-osteosynthesis surgical treatment must be considered since the mortality rate after 1 year of treatment is significant. Another critical point is the cognitive bias in older patients, as we did not apply specific instruments for cognitive screening over the telephone.

Our study also has strengths, as the practicality of the connection avoids unnecessary contact and facilitates the recruitment of the general population for epidemiological studies with no impact of social distancing situations such as the COVID-19 pandemic. The telephone use of DN4i also allows studies comparing clinical outcomes in different regions.

Future studies are potentially required to investigate whether telephone DN4i predicts treatment response since subjects with higher scores may be more responsive. It is also relevant to develop epidemiological studies to better detail these data in the literature, considering the greater practicality of telephone interviews. Research in hospitals analyzing the number of operated patients who developed neuropathic pain is also plausible for adjustments in given therapeutic approaches and management.

## Conclusion

The application of the DN4i questionnaire by telephone compared to the in-person application of the DN4 questionnaire shows strong agreement between individual items of the tools and the total score obtained.
