# Supplementary material for: Comparison of DN4 and DN4 Interview Tests in the Identification of Neuropathic Pain after Fracture Surgery
Source: Rev Bras Ortop (Sao Paulo). 2024 Mar 21;59(1):e125–9. doi: 10.1055/s-0044-1779686 (PMC10957271; doi:10.1055/s-0044-1779686)
Supplement: Related texts

# Research article: Comparação entre os testes DN4 e DN4 interview na identificação de dor neuropática após cirurgia de fraturas

**Keywords:** dor, entrevistas por telefone, fixação de fratura, inquéritos e questionários, síndrome da dor regional complexa

## Abstract

**Objetivo:** Comparar os resultados obtidos através do questionário DN4 com os do DN4i com aplicação via telefônica na identificação de dor neuropática após cirurgia de fraturas.

**Métodos:** Este foi um estudo metodológico com questionário aplicado presencialmente (DN4) e por telefone (DN4i). Foram elegíveis os participantes maiores de 18 anos de idade submetidos à cirurgia de fraturas em um hospital universitário no período de janeiro de 2017 a julho de 2020, que assinaram o Termo de Consentimento Livre e Esclarecido (TCLE) e puderam comparecer ao departamento de Ortopedia e Traumatologia do hospital. A concordância entre a pontuação total obtida na aplicação presencial e por telefone foi avaliada por meio do coeficiente de correlação de Pearson. O coeficiente de Kappa foi utilizado para avaliar a concordância entre os itens individuais dos questionários.

**Resultados:** Dos 53 participantes, 50 apresentaram resultado igual para rastreio de dor neuropática em relação ao DN4 com o DN4i, sendo 41 com escore positivo para dor neuropática e 12 com escore negativo. O coeficiente de correlação de Pearson e o coeficiente Kappa apresentaram r = 0.84.

**Conclusão:** O uso telefônico do DN4i comparado à aplicação presencial do DN4 apresenta forte concordância tanto entre os itens individuais dos questionários quanto entre a pontuação total obtida.

## Introdução

A dor neuropática é denominada como “dor que surge como consequência direta de uma lesão ou doença que afeta o sistema somatossensorial”. 1 Dentre os fatores associados à dor neuropática estão sexo feminino e idade avançada. 2 Estudos recentes também relatam que fratura e cirurgia são uma etiologia comum para o desenvolvimento de dor neuropática. 3 4

Dados epidemiológicos sobre dor neuropática ainda não estão completamente detalhados na literatura, mas evoluíram nos últimos anos. 5 No Brasil, estima-se uma prevalência de 14,5% a respeito de pacientes com dor crônica com características neuropáticas. 6

Dentre as ferramentas para rastreio de dor neuropática, o questionário *Douleur Neuropathique 4* (DN4) apresenta maior praticidade em relação aos demais testes por ter um número menor de itens e alta capacidade de discriminar dor neuropática de dor nociceptiva. 7 8 O DN4, desenvolvido pelo grupo francês de dor neuropática, contém 10 itens respondidos em sim ou não, em que o escore igual ou maior a 4 corresponde à presença de dor neuropática. 9 Também é possível aplicar o DN4 *interview* (DN4i) que engloba apenas os 7 itens iniciais, em que o escore positivo é obtido com pontuação igual ou maior a 3. 9 Santos et al. 7 validaram e traduziram o DN4 para o português brasileiro.

Ferramentas de rápida execução e alta sensibilidade para detecção de dor neuropática, utilizadas de forma presencial ou não, são viáveis para construção detalhada da epidemiologia e da etiologia, escolha adequada das intervenções terapêuticas e definição de prognóstico. Portanto, o objetivo do estudo foi comparar os resultados obtidos através da aplicação presencial do questionário DN4 com os do DN4i com aplicação via telefônica na identificação de dor neuropática após cirurgia de fraturas.

## Materias e Métodos

Trata-se de um estudo metodológico realizado a partir da verificação de prontuários de pacientes submetidos à cirurgia de fraturas em nosso hospital, considerando o intervalo de tempo de janeiro de 2017 a julho de 2020. O estudo recebeu aprovação ética do Comitê de Ética em Pesquisa (CAAE: 28504919.5.0000.5134).

Foi uma amostra por conveniência em que houve o recrutamento de todos os pacientes que se enquadravam nos critérios de elegibilidade, sendo pacientes maiores de 18 anos submetidos à cirurgia de fraturas em nosso hospital, que assinaram o Termo de Consentimento Livre e Esclarecido (TCLE) e poderiam comparacer ao departamento de Ortopedia e Traumatologia do hospital. Foram excluídos os pacientes que se rejeitaram a participar do estudo, não atenderam ao telefonema ou falecidos.

Um grupo treinado previamente de ortopedistas do corpo clínico do hospital aplicou presencialmente o DN4 validado e traduzido para português brasileiro aos pacientes. Após um período de 4 a 6 meses, duas pesquisadoras, que não tinham conhecimento dos resultados obtidos pelo grupo de ortopedistas, aplicaram o DN4i por telefone aos respectivos pacientes. Todos os dados coletados foram organizados em uma planilha Excel para análise posteriormente.

O DN4 é dividido em duas partes, sendo a primeira como entrevista com 7 itens e a outra como exame sensorial com 3 itens, enquanto o DN4i contém somente a parte em forma de entrevista, o que possibilita a autoaplicação pelo paciente ou uso telefônico. A parte com os 7 itens engloba 2 domínios, em que o primeiro avalia a característica da dor (queimação, sensação de frio dolorosa e choque elétrico) e o segundo os sintomas associados a sensações anormais na mesma área (formigamento, alfinetada e agulhada, adormecimento e coceira). A segunda parte do DN4 composta pelo exame sensorial aborda hipoestesia ao toque, hipoestesia à picada de agulha e dor causada ou aumentada por escovação. Cada item de ambos os questionários é respondido por meio de “sim” equivalendo a 1 ou “não” a 0. A soma das pontuações pode variar de 0 a 10 considerando o DN4, e de 0 a 7 no DN4i. O escore positivo para presença de dor neuropática é dado pela soma ≥ 4 no DN4 e ≥ 3 no DN4i. 9

As análises foram realizadas por um investigador independente. As estatísticas descritivas foram calculadas para caracterização da amostra, considerando todas as variáveis coletadas. Esses dados foram apresentados utilizando medidas de tendência central (média/mediana) e de dispersão (desvio padrão) para as variáveis quantitativas, e de frequência e percentual para variáveis categóricas.

Para investigar a concordância entre a pontuação total obtida no questionário, aplicado presencialmente (DN4) e por telefone (DN4i), foi utilizado o coeficiente de correlação de Pearson. Considerando os dados estatisticamente significativos, a magnitude do coeficiente de correlação foi interpretada da seguinte forma: forte (r≥0.6), moderado (0.3≤ r ≤0.59) e fraco (r≤0.29). 10

Foi utilizada a estatística ponderada κ para investigar a concordância entre os itens individuais dos questionários. Os coeficientes Kappa foram interpretados da seguinte forma: excelente (> 0.80), substancial (> 0.60), moderado (0.40-0.60), e razoável a pobre (< 0.40). 11 Todas as análises foram realizadas utilizando o Software estatístico SPSS 17.0 para Windows, com um nível de significância de 5%.

## Resultados

No total, 71 participantes foram elegíveis para o estudo. A amostra resultou em 53 participantes com dados analisáveis, dos quais 50 (94.3%) apresentaram resultado igual para rastreio de dor neuropática em relação ao DN4 com o DN4i. A disposição da amostra é apresentada na Fig. 1 .

**Fig. 1:** Recrutamento da amostra de acordo com os critérios de elegibilidade.

As características sociodemográficas e clínicas dos participantes com dados analisáveis estão dispostas na Tabela 1 . Considerando a Escala Verbal Numérica de dor que varia de 0 a 10, 26.4% dos participantes relataram valores entre 2 e 8, em que metade (13.2%) apresentou escore positivo no DN4i.

**Tabela 1:** Características sociodemográficas e clínicas da amostra (n = 53)

Presença de dor neuropática (n = 12)	Ausência de dor neuropática (n = 41)
Sexo feminino (n, %)	33 (62.3)
Idade, média (±DP)	55.5 (± 17.7)
IMC, média (±DP)	27.95 (±5.52)
Presença de comorbidade (n, %)	33 (62.3)
Diabetes	11 (33.3)
Estado Civil (n, %)	
Solteiro	21 (39.6)
Casado	18 (34.0)
Viúvo	10 (18.9)
Divorciado	1 (1.9)
Não informado	3 (5.7)
Região de fratura (n, %)	
Tornozelo	23 (43.4)
Rádio	19 (35.8)
Fêmur proximal	11 (20.8)
Mecanismo de trauma (n, %)	
Queda da própria altura	23 (43.4)
Entorse	16 (30.2)
Queda de altura	7 (13.2)
Acidente de trânsito	6 (11.3)
Agressão	1 (1.9)
Escala Verbal Numérica de dor (n, %)	
Presença de dor	14 (26.4%)
Escore positivo no DN4i	7 (13.2%)

O grupo de ortopedistas que realizou a aplicação pessoalmente do DN4 obteve 13 positivos (soma ≥ 4) e 40 escores negativos, enquanto as duas pesquisadoras que efetuaram as ligações telefônicas alcançaram 12 positivos (soma ≥ 3) e 41 resultados negativos. A comparação das porcentagens das respostas positivas aos itens do DN4 e DN4i são apresentadas na Tabela 2 .

**Tabela 2:** Comparação da frequência das respostas positivas aos itens do DN4 e DN4i (n = 53)

Entrevista presencial (DN4)	n (%)
Queimação	15 (28.3)
Sensação de frio dolorosa	5 (9.4)
Choque elétrico	9 (16.9)
Formigamento	14 (26.4)
Alfinetada e agulhada	12 (22.6)
Adormecimento	10 (18.9)
Coceira	6 (11.3)
Hipoestesia ao toque	8 (15.1)
Hipoestesia à picada de agulha	4 (7.5)
Escovação	10 (18.9)
Entrevista por telefone (DN4i)	9 (16.9)
Queimação	8 (15.1)
Sensação de frio dolorosa	8 (15.1)
Choque elétrico	15 (28.3)
Formigamento	8 (15.1)
Alfinetada e agulhada	11 (20.8)
Adormecimento	
Coceira	6 (11.3)

O coeficiente de correlação de Pearson apresentou r = 0.84 (p < 0.001) para a concordância entre a pontuação total obtida nos questionários, o que é interpretado como forte considerando a magnitude do coeficiente de correlação. Referente à concordância entre os itens individuais dos questionários, o coeficiente Kappa obteve r = 0.84 (p < 0.001) sendo considerado como excelente.

## Discussão

Observamos em nosso estudo que dos 12 resultados positivos no DN4i, 7 eram mulheres, das quais 4 tinham diabetes mellitus. Esse achado reforça os fatores associados ao desenvolvimento de dor neuropática, como sexo feminino e diabetes mellitus como comorbidade associada. 2 12 13

Em relação ao sítio da fratura, houve predominância de fraturas de tornozelo (n = 23), em que 17.4% tiveram escore positivo no DN4i. Rbia et al. 14 reforçam esse achado, identificando prevalência de dor neuropática após cirurgia de fratura de tornozelo em 23% dos 271 pacientes, o que ocasionou impacto na qualidade de vida.

Attal et al. 15 sugerem o uso do DN4i por médicos e demais profissionais de saúde para triagem rápida de dor neuropática. Bouhassira et al. 9 haviam sugerido em estudo prévio que o DN4i poderia ser utilizado em pesquisas telefônicas devido às significativas propriedades discriminantes dos 7 itens. Essa observação foi embasada no fato do DN4i apresentar sensibilidade (78%) e especificidade (81.2%) ligeriramente menores ao DN4 (82.9% e 89.9%, respectivamente). 9 16 Contudo, VanDenKerkhof et al. 17 apontam que a sensibilidade do teste pode variar de acordo com a condição clínica, por exemplo, citaram sensibilidade de 92.5% para dor central enquanto Aho et al. 18 relataram 66.2% para lesão de nervo periférico após procedimento cirúrgico.

Apesar da sugestão do uso telefônico por Bouhassira et al., 9 poucos estudos na literatura analisaram o uso telefônico do DN4i. Em um deles, 16 a validação foi durante uma pesquisa telefônica complementar, a qual não foi referenciada, para determinar a prevalência de dor crônica com sintomas de dor neuropática em uma amostra aleatória da população de Alberta, Canadá. Os participantes da pesquisa receberam a ligação uma semana após a aplicação presencial do DN4, o que favorece o viés de memória, dado que o paciente poderia lembrar as respostas e repeti-las. Além disso, a ligação foi executada pelo mesmo médico que aplicou o DN4, havendo comprometimento da validade interna e externa do estudo. Enquanto em nosso estudo, a aplicação presencial do DN4 e via telefônica do DN4i foi realizada por indivíduos diferentes, mas com o mesmo treinamento, e considerou um tempo maior de intervalo (4-6 meses) para controle do viés de memória.

Keene et al. 19 corroboram que o uso do questionário DN4i via telefônica, meses após o DN4, não diminui a sensibilidade para rastreio de dor neuropática de acordo com seu estudo multicêntrico para identificar a prevalência de dor neuropática após cirurgia de fratura de membro inferior através da aplicação do DN4 no terceiro e sexto mês. Como resultado, observaram que 10% dos pacientes sem dor neuropática com 3 meses de cirurgia apresentaram a dor aos 6 meses, o que difere da expectativa que a intensidade e o caráter da dor melhoram com o tempo pós-lesão. 19

O estudo apresenta algumas limitações como a dificuldade em contactar pacientes operados há mais tempo, o que limitou a quantidade de participantes. Por incluir fraturas de fêmur, deve-se considerar o viés de mortalidade em tratamento cirúrgico pós osteossíntese, dado a taxa de mortalidade significativa após 1 ano de tratamento. Outro ponto importante é o viés cognitivo em pacientes com idade mais avançada, pois não aplicamos instrumentos específicos para rastreio cognitivo por telefone.

Nosso estudo também apresenta pontos fortes, pois a praticidade da ligação evita o contato desnecessário e facilita o recrutamento da população em geral para estudos epidemiológicos, sem ser afetado por situações de distanciamento social como a pandemia da COVID-19. O uso telefônico do DN4i também possibilita a realização de estudos que comparem desfechos clínicos em diferentes regiões.

Estudos futuros podem ser necessários para investigar se o DN4i por telefone é preditivo para a resposta ao tratamento, tendo em vista que indivíduos com escores mais altos podem ser mais responsivos. Também é relevante o desenvolvimento de estudos epidemiológicos para melhor detalhamento desses dados na literatura, considerando a maior praticidade da entrevista por telefone. Pesquisas em hospitais que analisem a quantidade de pacientes operados que desenvolveram a dor neuropática também são plausíveis para ajustes em determinadas condutas terapêuticas e gestão.

## Conclusão

A aplicação do DN4i por telefone comparada à presencial do DN4 apresenta forte concordância tanto entre os itens individuais dos questionários quanto entre a pontuação total obtida.
